# Use of nano-scale synthesized polyurethane acrylate as a binder in textile printing

**DOI:** 10.1038/s41598-026-42613-w

**Published:** 2026-03-26

**Authors:** Karima M. Haggag, Mohamed M. El-Molla, Fatma N. El-Shall, Ahmed I. Hashem

**Affiliations:** 1 Textile Research and Technology Institute, National Research Centre, El- Bohouth St., Dokki, P.O.12622, Giza, Egypt; 2 Chemistry Department, Faculty of Science, Ain Shams University, Abbassia, Cairo, 11566 Egypt

**Keywords:** Binders, Polyurethane, Pigment Printing, Synthesized, Textile fabrics, Chemistry, Materials science

## Abstract

Four Nano-Scale synthesized polyurethane acrylates were utilized in pigment printing as a binder for screen printing of different textiles. The study of the impact of time, temperature, binder concentration, and thermos-fixation curing on color strength and print durability of cotton, polyester, and cotton/polyester fabrics was conducted. The findings demonstrated that the fabricated polyurethane acrylate binders could effectively secure pigments to cotton, polyester, and cotton/polyester blends using thermos-fixation. Overall, the prints produced with these binders exhibited superior color strength compared to commercial binders. Printed cotton fabrics exhibit acceptable rubbing for both wet and dry results, with all binders used. But printed polyester and CO/PET fabrics show enhanced dry rubbing but lower wet rubbing fastness results, depending on the type of binder used. The K/S values are 13.8, 12.2, 14.8, and 13, using binder with a concentration of 5 g /L in printing paste, PUAmcs, PUAms, PUAm, and PUAt, respectively, compared to the commercial binder with a concentration of 50 g /L in printing paste at the same conditions, the K/S is 12.8. Both the washing and perspiration fastness ranged from very good to excellent for all, and the dry rubbing for all binders used ranged from good to very good. The effect of increasing the thermal fixation temperature from 80 to 140 °C leads to an increase in the K/S, and after that, increasing the temperature to 160 °C, produced a slight increase in K/S values. This is true irrespective of either the concentration or the type of binder used. Binder PUAmcs seem to have produced prints with the highest K/S values as compared with the results obtained by the commercial binder and binders (PUAms, PUAm, and PUAt) in most cases. As observed in the SEM images, the binding agents applied to the surface of the printed textiles were able to generate a homogeneous and uniform coating layer.

## Introduction

The difference between pigment and dye coloration of textile fabrics from a textile process point of view is that dyes are colored chemical compounds containing reactive groups able to react with certain functional groups within the specified fabric. The coloration occurred through a chemical reaction between dye and fabric under the required conditions^1,2^. However, there are many limitations for the dyes to be used in a wide range of textile coloration such as a limited color range, specificity of dye application, poor light fastness, and extremely non-economic processing, etc^3^. Printed materials largely rely on pigment printing, accounting for over 80% of the production. This method offers clear benefits, including versatility and the ability to achieve near-final print results during the printing process^4^. The pigment compounds consist solely of color molecules, lacking the requirement for reactive or functional groups, as well as any affinity for fiber. Their interaction and adhesion with the fabric occurred with the aid of a polymer compound called a binder. Many favorable features, such as applying to most fabric types^5^, characterize the use of binders in the coloration process. The binder is a film-forming element that fixes the pigment or dye particles in a homogeneous system and forms a film holding these colored particles inside after the drying and curing stages. It is commonly a polymer or copolymer of unsaturated monomers like acrylate compounds, styrene, and acrylonitrile, etc^6^.

Despite these progresses, there are many drawbacks to the commercial polymeric binders that are presently available for pigment printing. Durability issue: A wide range of binders have poor abrasion and washing resistance, resulting in color fading and shorter textile life. Environmental Impact: Conventional binders, which are mostly petroleum-based, are unsustainable since they are not biodegradable and emit high levels of volatile organic compounds. Limitations on Flexibility: Because they lack the elasticity necessary for flexible fabrics, several binders stiffen after curing. Color Clarity: Over time, the visual quality of current binders may decrease, resulting in yellowing or a drop in pigment intensity. Processing Efficiency: Extended drying times and high curing temperatures increase energy usage and production fees. These limitations highlight the need for synthetic binders for improving processing efficiency, flexibility, durability, color accuracy, and environmental friendliness.

The printing system consists of three basic components: pigment dispersion, thickeners and auxiliary agents, and polymeric binders and crosslinking agents, which work together to produce the required print quality. Polymers must possess specific characteristics to enable them to serve as binders in pigment printing coloration processes. They should be colorless, have low viscosity, and be stable in atmospheric conditions, and their structures should include reactive groups capable of further reaction under curing conditions to produce a desired film (traded pigment particles inside them)^2,7^. Moreover, Polyurethane polymers are the most important class of polymers with brilliant properties to be applied in coating, adhesion, and textile finishing processes^8^.

Multiple studies have been conducted to improve the features of bonding-agent materials. Waterborne polyurethanes (WPU) are less hazardous and ecologically benign and are widely employed in textile applications. Hyper-branched polyurethanes (HPUs), with their numerous reactive end groups, compact molecular structure, and reduced chain entanglement, exhibit distinctive characteristics such as high solubility, reactivity, and desirable rheological behavior. Adding chitosan, oil, and nanometals such as Ag, Cu, and Zn can increase the stiffness and mechanical strength of WPU coatings, in addition to their antibacterial qualities, as observed during their application in fabric finishing. Nano-emulsion co-polymer particles were prepared using various butyl methacrylate/acrylic acid (BMA/ACA) ratios and used as a binder in textile pigment printing paste with high fastness qualities^9–11^. Waterborne polyurethane acrylate/zinc oxide NP systems have been designed to be utilized as multifunctional binding agents when printing mixed textiles with pigment colors. The prepared composites (PUA/ZnO) were effectively used as binders, resulting in improved color intensity and fastness. Furthermore, printed materials provide exceptional UV protection and antimicrobial qualities^12^. However, pigment coloration has some industrial and ecological problems, such as relatively high-temperature cure, stiff hand, poor crock fastness, formaldehyde emissions, and clogging nozzles and screens in both textile inkjet and screen printing processes. These disadvantages are related to the binding agent. Thus, to improve the quality of the textile pigmented colored goods, the overall properties of the binding agents should be improved^13^.

The present work was carried out with the following objectives: use of nano-scale synthesized polyurethane acrylate as an aqueous binder, which is described in the reference^13^, as a binding material in pigment printing of cotton, polyester, and their blend fabrics by the silk screen technique using pigment colors.

### Materials

#### Fabric

100% Scoured, bleached cotton fabric plain weave (140 g/m^2^), 100% white polyester fabric plain weave (149 g/m^2^), and cotton/polyester blend CO/PET 50/50% fabric (135 g/m^2^) were supplied by Misr Company for Spinning and Weaving, Mahala El–Kubra, Egypt.

#### Pigment

Green 3GL pigment (commercial grade) supplied by Daico Chemical Industry, Cairo, Egypt.

#### Printing auxiliaries

Ammonium persulfate (NH₄)₂S₂O₈ (Merck-Germany- (reagent grade, 98%) as thermal initiator. Bercolin CPK, supplied by Berssa-Turkey, is a thickening agent (commercial grade). Bercolin metal CM supplied by Berssa-Turkey, as a Thermal curing binder (commercial grade). N, N-dimethyl formamide (DMA) (reagent grade, 99.9%) from ACROS Chemical Co.

### Methods

#### Preparation of Binder

Four Nano-Scale synthesized polyurethane acrylate binders having different contents of vinyl groups were prepared as illustrated in^12,13^. In a modified one-necked flask inside a Milestone MW reactor (2.45 GHz, 1200 W max)— or a three-necked flask equipped with a stirrer, thermometer, and reflux condenser under a nitrogen atmosphere and heating oil bath (Thermal Heating)—PPG (2000 g/mol) and sorbitol were dissolved in N, N-dimethylacetamide (DMAc) solvent (stirred at 60 °C and 200 W for 10 min). Temperature and MW power were reduced to 40 °C and 100 W. Isophorone diisocyanate (IPDI), with or without 0.05% (w/w) DBTDL catalyst, was slowly added over 5 min. The mixture was stirred at 60 °C and 200 W for 10 min. Temperature and power were adjusted to 40–50 °C and 150 W. Hydroxyethyl acrylate (HEA) was gradually added. The molar ratios of one mol of PPG with three mol of IPDI, and two mol of HEA).

- PUAmcs prepared using microwave irradiation with the aid of a catalyst and solvent.
- PUAms, prepared using microwave irradiation with the aid of a solvent only.
- PUAm was prepared using microwave irradiation without a catalyst or solvent.
- PUAt prepared conventional thermal heating.

Microwave-synthesized polyurethane acrylate oligomers and that of the thermally synthesized one (PUAt), it is observed that all microwave-synthesized polyurethanes show a smaller particle size, regular and uniform particle structure, with a narrower distribution compared to PUAt particles. In addition to this feature, all PUAm appear to be in a nanoscaled size with particle sizes in the range between 37 and 17 nanometers^13^.

#### Printing

##### Preparation of printing pastes

The pigment printing pastes were prepared according to the following recipe.

**Table:** 

Pigment	50 g/L
Binder*	X g/L
Thickener	40 g/L
Initiator**	10 g/L
Distilled water	Y

* The binders’ concentrations were (5, 10, 15, 20, and 25 g/L).

The commercial binder was used at a concentration of 50 g/L. It is used for comparing the results with those obtained.

#### Printing techniques

Printing paste containing binder with selected concentration, the thermal initiator (ammonium persulphate), in addition to the other ingredients was prepared. The homogenized printing pastes were applied to the fabrics using a flat-screen printing technique.

#### Pigment fixation

Different temperatures (80,100,120-and160°C) for periods of 4, 6, and 8 min in a thermo-fixation-instrument were applied to printed fabrics.

### Measurements and analysis

#### Scanning electron microscope (SEM)

Selected printed cotton fabrics, samples printed, dried, and then cured at the recommended fixation conditions (160 °C and 4 min). After curing, the samples were washed with water to remove any unfixed pigment particles from the fabric surface. Printed cotton fabrics mounted on aluminum stubs, and sputter-coated with gold in a 150 Å sputter (Coated Edwards) and examined by Jeol (JXA-840 A) Electron Probe Microanalysis (Japan), magnification range 35 − 10,000, accelerating voltage 19 kV^14,15^.

#### Color strength measurements

The relative color strength of the prints expressed as the K/S^16^ value of the colored samples was determined by reflection measurements using the Data Color International model SF 500, USA.

#### Fastness properties

Fastness to washing was measured according to the Launder-O-meter AATCC Test method 61–2013. Rubbing and perspiration measurements were done according to the standard methods of AATCC 1993b and AATCC 1993c, respectively^17,18^.

## Results and discussion

After the printing process has been done on the fabric surface, the fixation or curing process (at specified higher temperatures where the reactive monomers cross-link and are converted to tough polymer) has been followed.

This process is needed for the polymerization of binder molecules together to produce a thin film that fixes the colored molecules inside it and prevents them from being released^19^. The generation of sulfate ions from persulfate groups, which initiates the crosslinking reaction in the curing stage, takes place in the presence of H_2_O and heat.
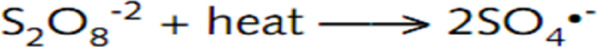


The radicals attack the reactive groups on polyurethane acrylate polymer to form reactive intermediates and the propagation reaction continues. This process will go on until the cross-linked polymer is produced as the formula clarified below in the following diagram or the reaction is terminated.
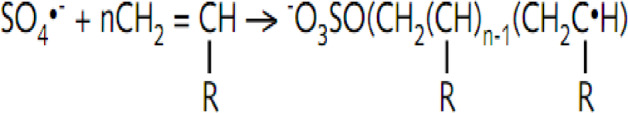


Using and evaluating the synthesized binders I-IV PUAmcs, PUAms, PUAm and PUAt) as binders in pigment printing pastes for printing cotton, polyester, and their blends, the results were compared with those obtained when a commercial binder was used.
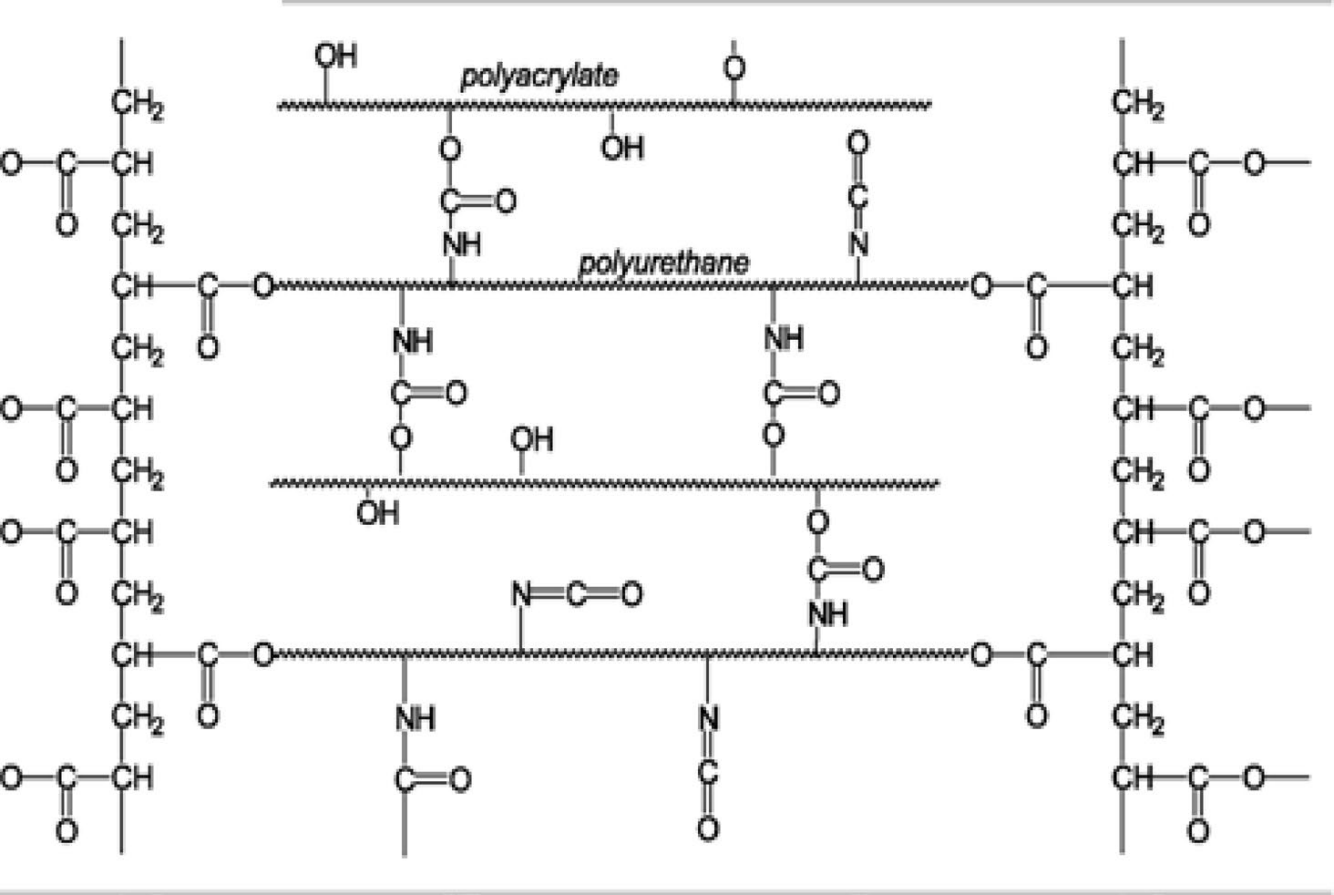


Diagram of Cross-linked three-dimensional polyurethane network^15^.

### Pigment printing of cotton fabrics

**Fig. 1:**
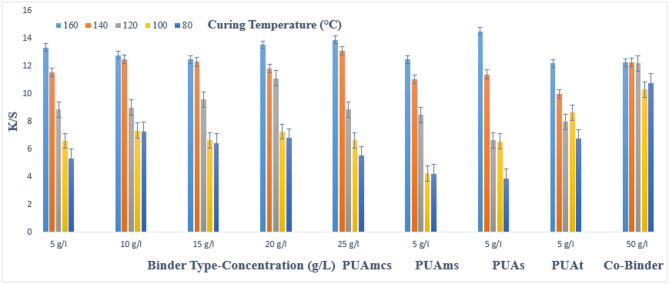
Effect of using different concentrations of binders (I-IV), on the K/S of printed, thermally* cured cotton fabrics for 4 min. *Curing temperatures 80, 100, 120, 140, and 160 °C.

**Fig. 2:**
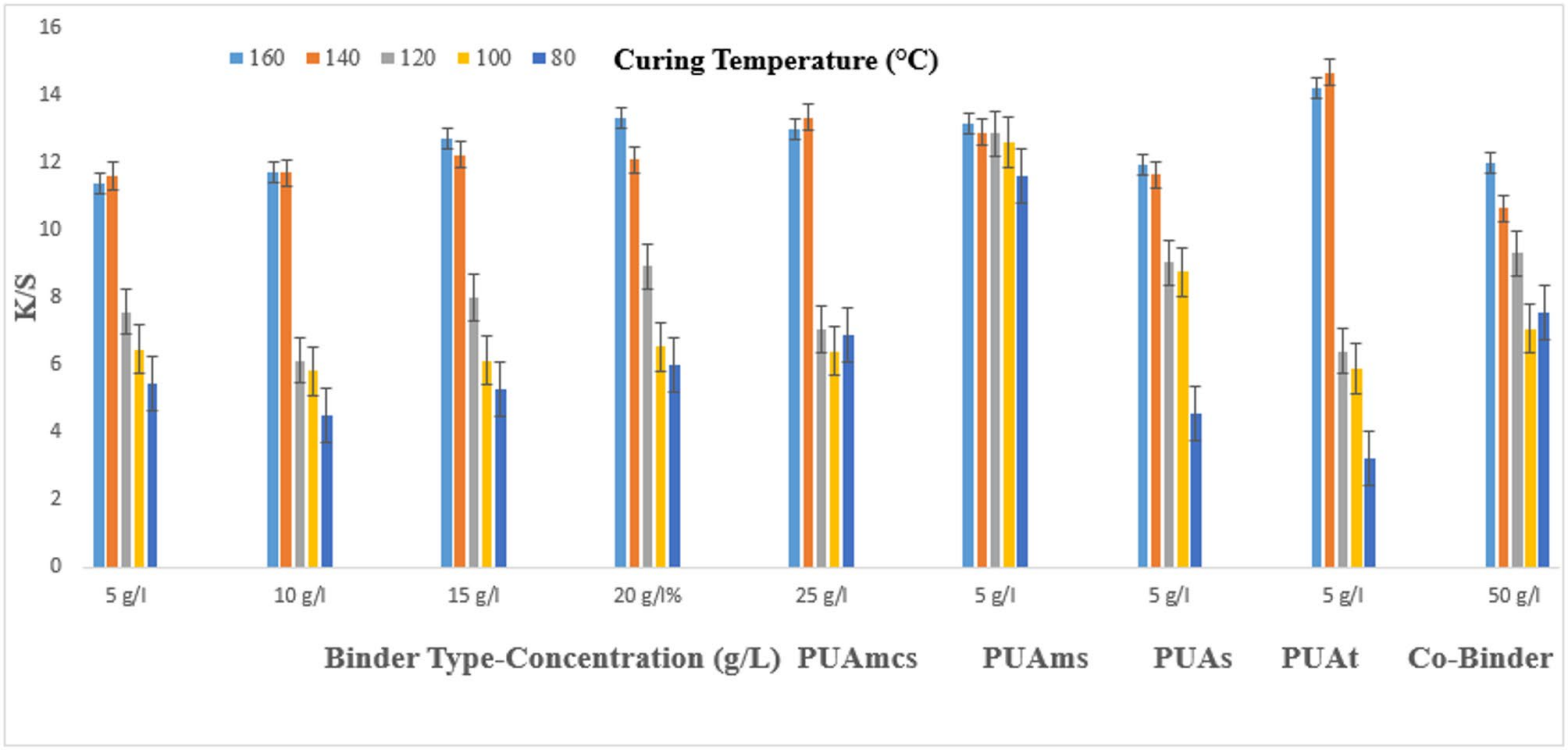
Effect of using different concentrations of binders (I-IV) on the K/S of printed, thermally* cured cotton fabrics for 6 min. *Curing temperatures 80, 100, 120, 140, and 160 °C.

**Fig. 3:**
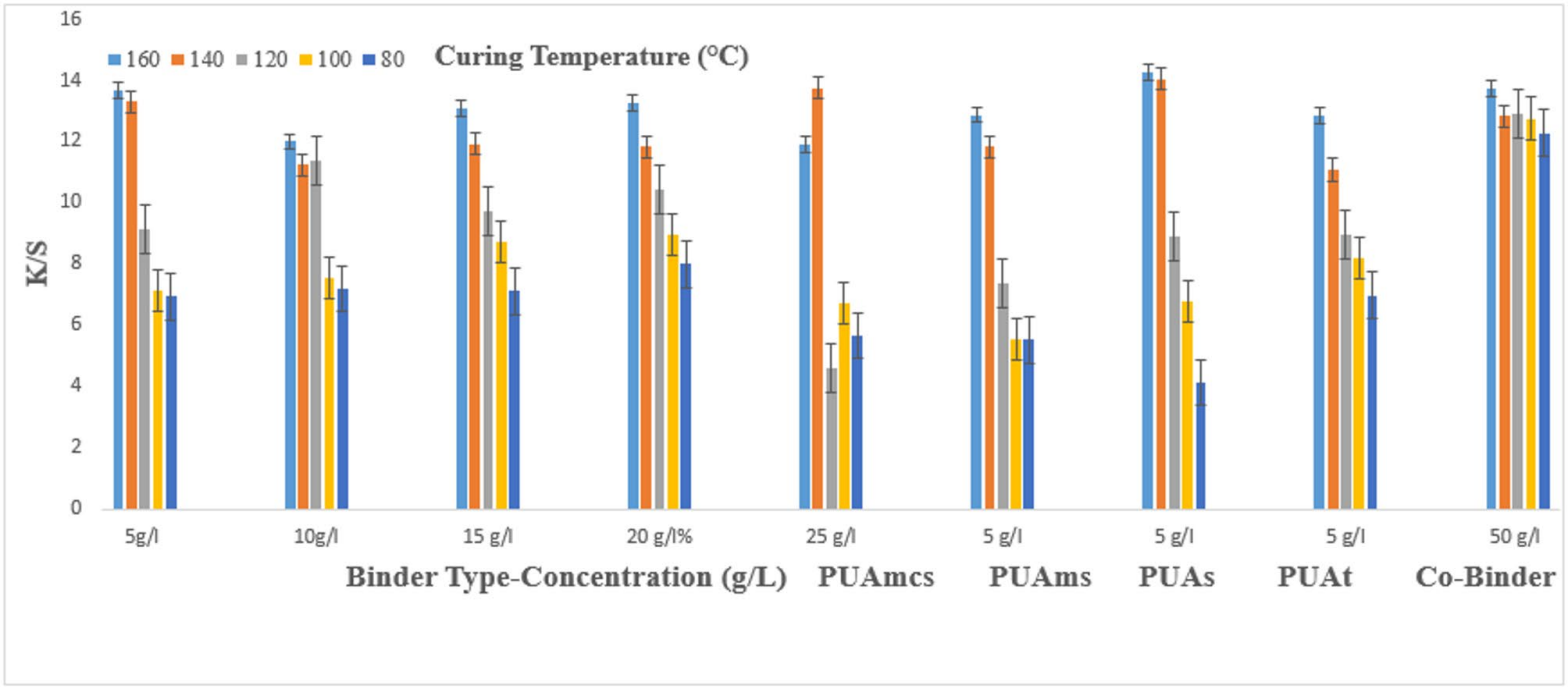
Effect of using different concentrations of binders (I-IV) on the K/S of printed, thermally* cured cotton fabrics for 8 min. *Curing temperatures 80 °C, 100 °C, 120 °C, 140 °C, and 160 °C.

The prepared pigment pastes, containing 5 g/l Green 3GL, using different concentrations (5, 10, 15, 20, and 25 g/L) of the synthesized binders I-IV, as well as the commercial binder (50 g/L, as the company recommended), were printed on cotton fabrics. The samples were dried and then cured at different temperatures (80, 100, 120, 140, and 160 °C) for 4, 6, and 8 min intervals times respectively. The (K/S) results of the pigment-printed cotton fabrics are represented by Figs. 1, 2 and 3. The Figures show the effect of using the aforementioned binders on the K/S of the printed cotton fabrics. It is clear from the results, that both the concentration and type of binders (I-IV) have some effects on the K/S value of the produced printed cotton fabrics.

It is clear that, upon using binder concentrations of 5 and 10 g/L in the printing paste, they produce comparable K/S results. Further increase in the binder concentration up to 25 g/L produced no significant color enhancement, and therefore it is not recommended for saving costs. From Figs. 1, 2 and 3. Synthesized Binders I-IV, produced cotton prints with comparable K/S values, in most cases.

In addition, it is noticed that, in some cases, higher K/S values are produced with cotton prints using binders (I-IV) compared with results obtained using commercial binders under the same conditions. For example, as shown in Fig. 1, and binder concentrations of 5 g/L in the printing paste, using PUAmcs, PUAms, PUAm and PUAt, at fixation temperature 160 °C and time of fixation 4 min., the K/S values is 13.8, 12.2, 14.8 and 13 respectively, compared to the commercial binder with concentration 50 g/L in printing paste at the same condition used the K/S is 12.8.

The effect of increasing the thermal fixation time from 4 to 8 min on the K/S values of the prints is also illustrated in Figs. 1, 2 and 3. A fixation time of 4 min. could be considered adequate under such conditions, and no need to prolong the time of fixation any further.

In addition, it is noticed that increasing the fixation temperature from 80 to 140 °C leads to an increase in the K/S values. After that, increasing the temperature to 160 °C, brings a slight increase in K/S values. This is true irrespective of either the concentration or the type of binder used. Therefore, it is also not recommended to use higher fixation temperatures over 140 °C to save energy. The optimum conditions of thermal fixation could be considered as 140 °C for 4 min.

### Pigment prints of polyester fabrics

The prepared pigment pastes, containing 5 g/L Green 3GL, and different concentrations (5, 10, 15, 20, and 25 g/L) of the synthesized binders I-IV (PUAmcs, PUAms, PUAm, and PUAt), as well as the commercial binder (50 g/L, as the company recommended) (Co-Binder), were printed on polyester fabrics. The samples were dried and then thermally fixation at different temperatures (80, 100, 120, 140, and 160 °C) for different times, 4, 6, and 8 min respectively.

**Fig. 4:**
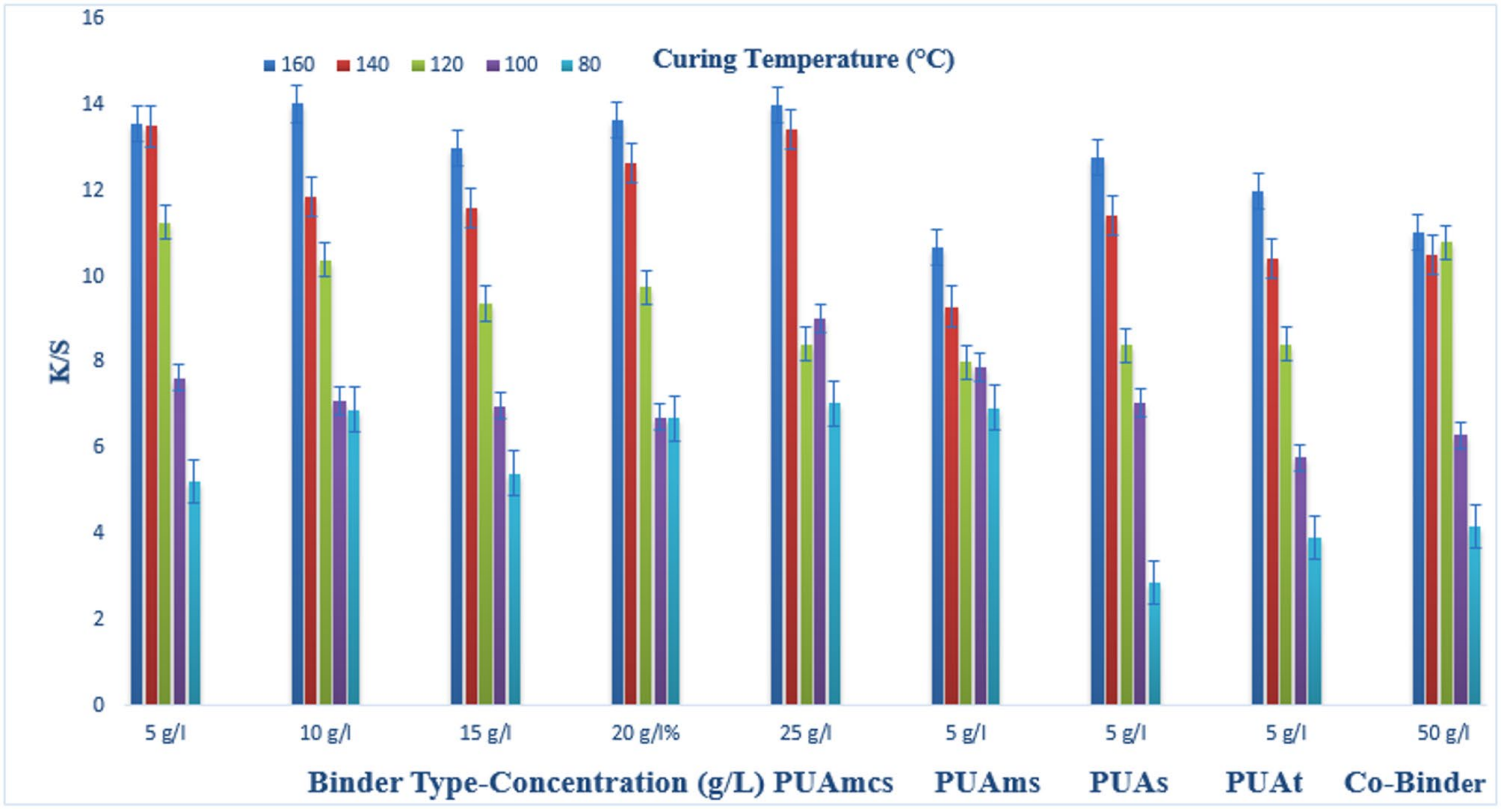
Effect of using different concentrations of binders (I-IV) on the K/S of printed, thermally* cured polyester fabrics for 4 min. *Curing temperatures 80, 100, 120, 140, and 160 °C.

**Fig. 5:**
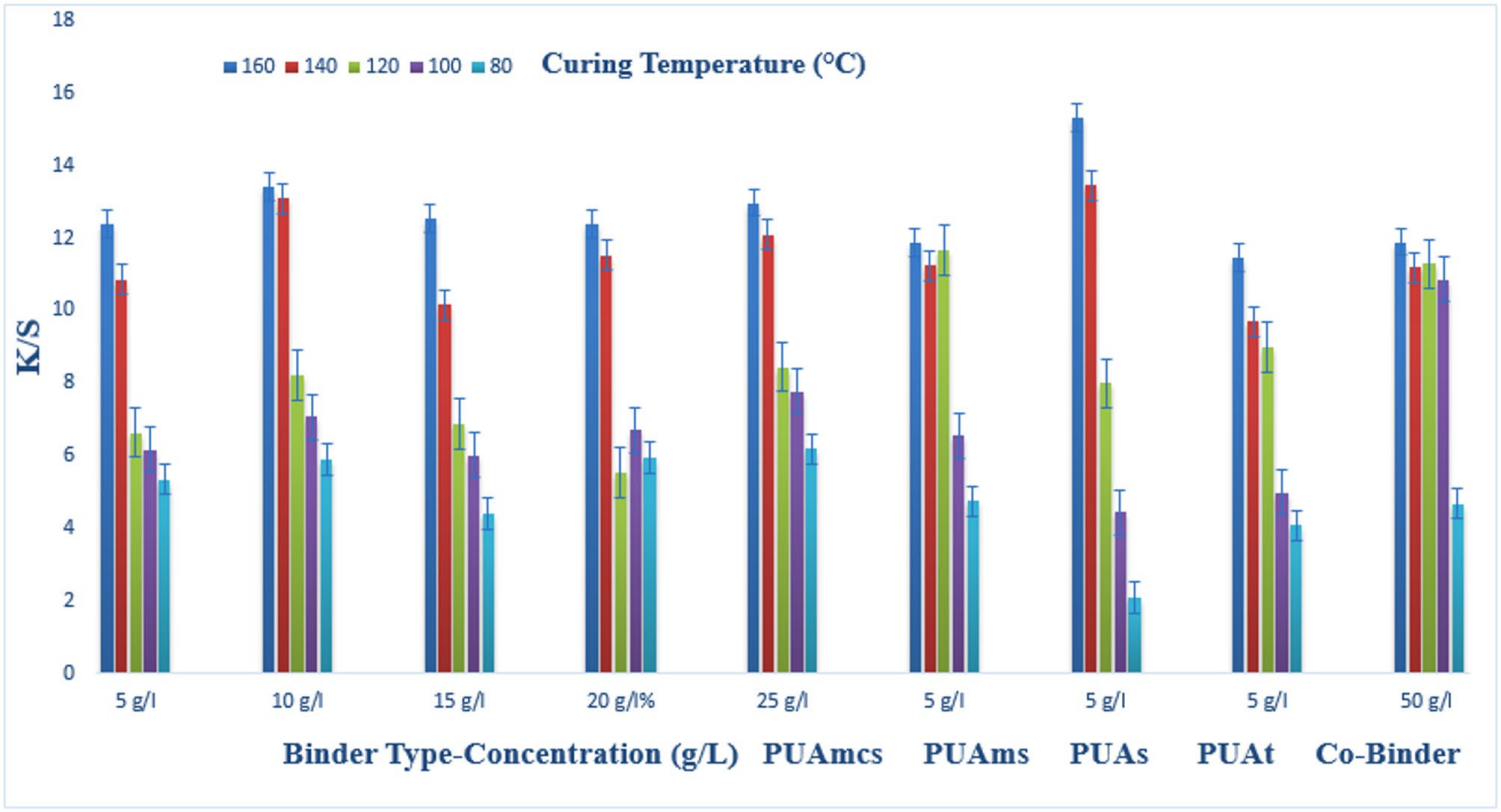
Effect of using different concentrations of binders (I-IV on the K/S of printed, thermally* cured polyester fabrics for 6 min. *Curing temperatures 80, 100, 120, 140, and 160 °C.

**Fig. 6:**
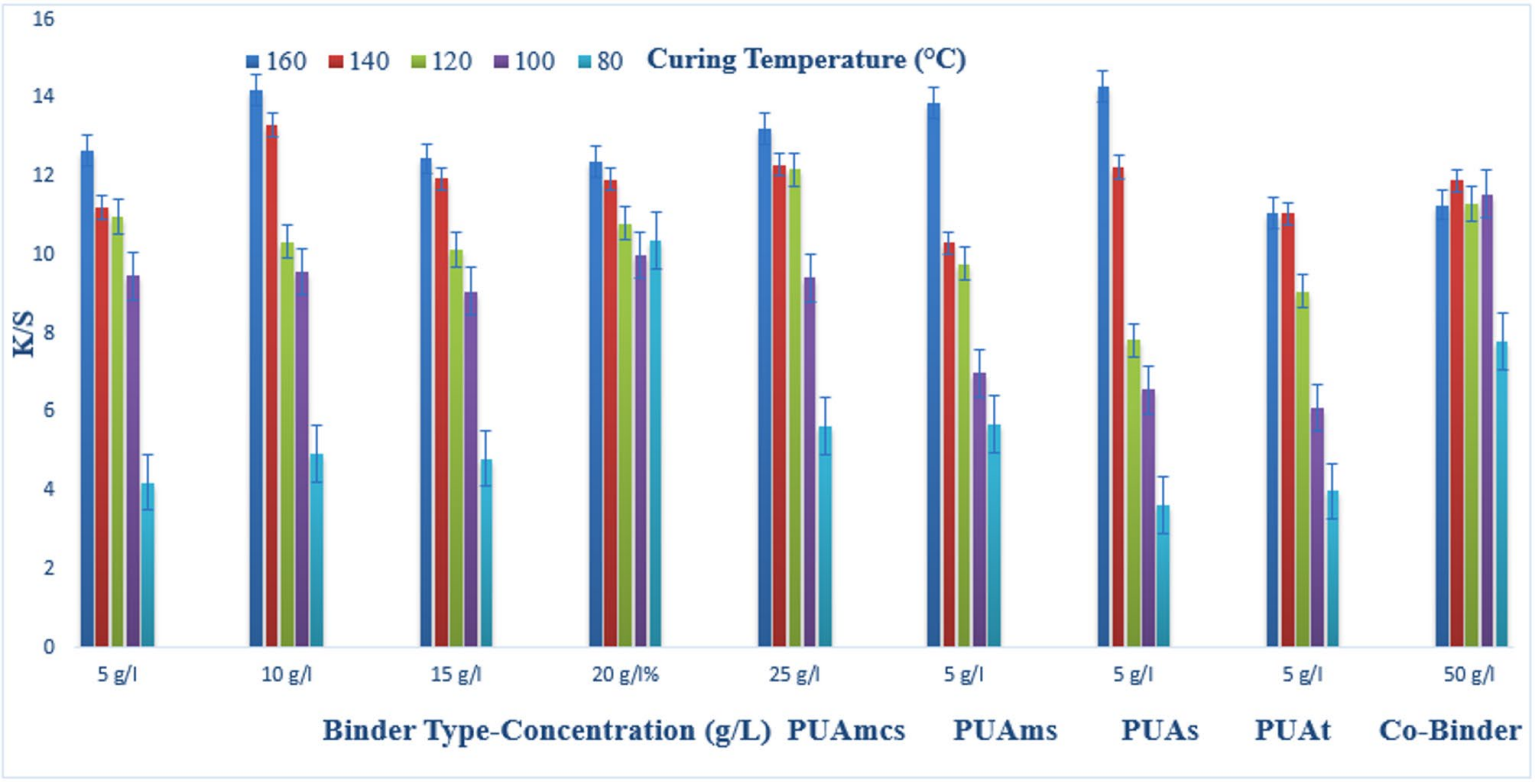
Effect of using different concentrations of binders (I-IV) on the K/S of printed, thermally* cured polyester fabrics for 8 min. *Curing temperatures 80, 100, 120, 140, and 160 °C.

Figures 4, 5 and 6 shows the effect of using binder concentration on the K/S of the printed polyester fabrics. From the figs, it is noticed that, upon using binder concentrations of 5 and 10 g/L in the printing paste, they produce comparable K/S results. Further increase in the binder concentration up to 25 g/L produced a slight color enhancement, and therefore, it is not recommended.

Also from the Figs. 4, 5 and 6, it is evident that synthesized binders (I-IV) produced polyester prints with comparable K/S values, in most cases.

As compared to polyester prints produced using binders (I-IV) with those produced using commercial binders, it was observed that, in some cases, higher K/S values are produced.

The effect of increasing the thermal fixation time from 4 to 8 min on the K/S results of the prints is also shown in Figs. 4, 5 and 6 respectively. A fixation time of 4 min could be considered an adequate time under such conditions, and no need to prolong the time of fixation any further.

Also, it is noticed that the effect of increasing the thermal fixation temperature from 80 to 140 °C leads to an increase in the K/S values. Further, an increase in the temperature to 160 °C brings a slight increase in K/S values irrespective of either the concentration or the type of binder used. Therefore, it is also not recommended to use higher fixation temperatures over 140 °C in this case. The optimum conditions of thermal fixation could be considered as 140 °C for 4 min. Lower binder concentrations ranging between 5 g/L – 10 g/L (binder I) PUAmcs are required to obtain prints with higher K/S values. Further increase in the binder concentration up to 25 g/L results in minor changes in the K/S of the polyester prints. The reverse is noticed with the commercial binder, with which higher concentrations of 50 g/L are needed to produce polyester prints with comparable K/S values to K/S obtained on using synthetic binders which affects the economic aspects of the process.

Generally speaking, binder PUAmcs seem to have produced prints with the highest K/S values as compared with the results obtained by the commercial binder and binders (PUAms, PUAm, and PUAt) in most cases. This could be attributed to the increase in the amount of vinyl groups, which leads to increased binder polymerization, resulting in better pigment fixation.

As shown in Fig. 4, For example, using binder concentrations of 5 g/L in the printing paste, using PUAmcs, PUAms, PUAm and PUAt, at fixation temperature 160 °C and time of fixation 4 min., the K/S values is 13.6, 11.6, 13 and 12 respectively, compared to the commercial binder with concentration 50 g/L in printing paste at the same condition used the K/S is 11.3.

### Pigment printing of cotton polyester blend (CO/PET) fabrics

The prepared pigment pastes, containing 5 g/L Green 3GL, and different concentrations (5, 10, 15, 20, and 25 g/L) of the synthesized binders (I-IV) (PUAmcs, PUAms, PUAm, and PUAt), as well as the commercial binder (50 g/L, as a company recommended), were printed on CO/PET fabrics by the flat screen technique. The samples were dried and then thermally fixation at different temperatures (80, 100, 120, 140, and 160 °C) for different 4, 6, and 8 min respectively.

**Fig. 7:**
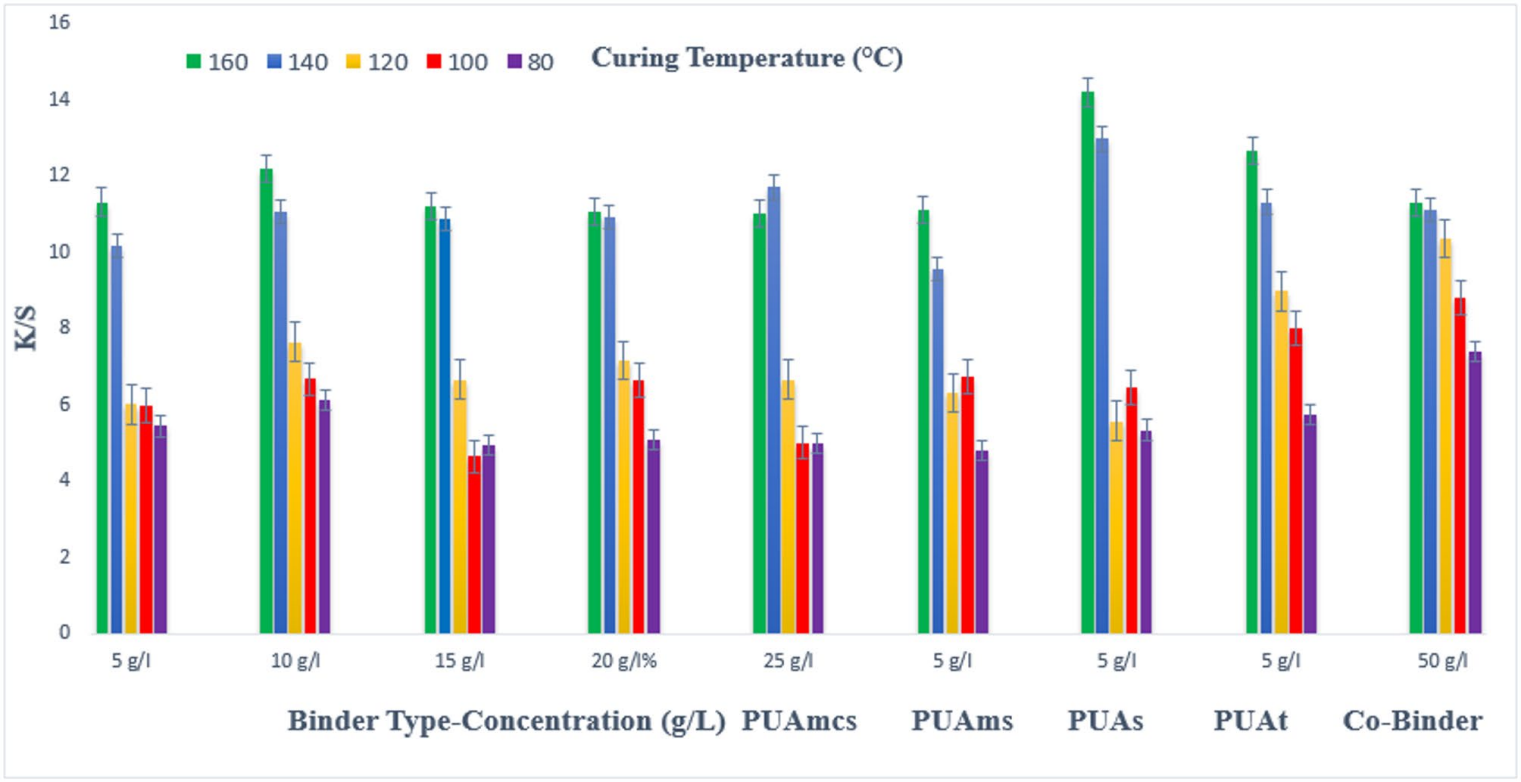
Effect of using different concentrations of binders I-IV on the K/S of printed, thermally* cured CO/PET fabrics for 4 min. *Curing temperatures 80, 100, 120, 140, and 160 °C.

**Fig. 8:**
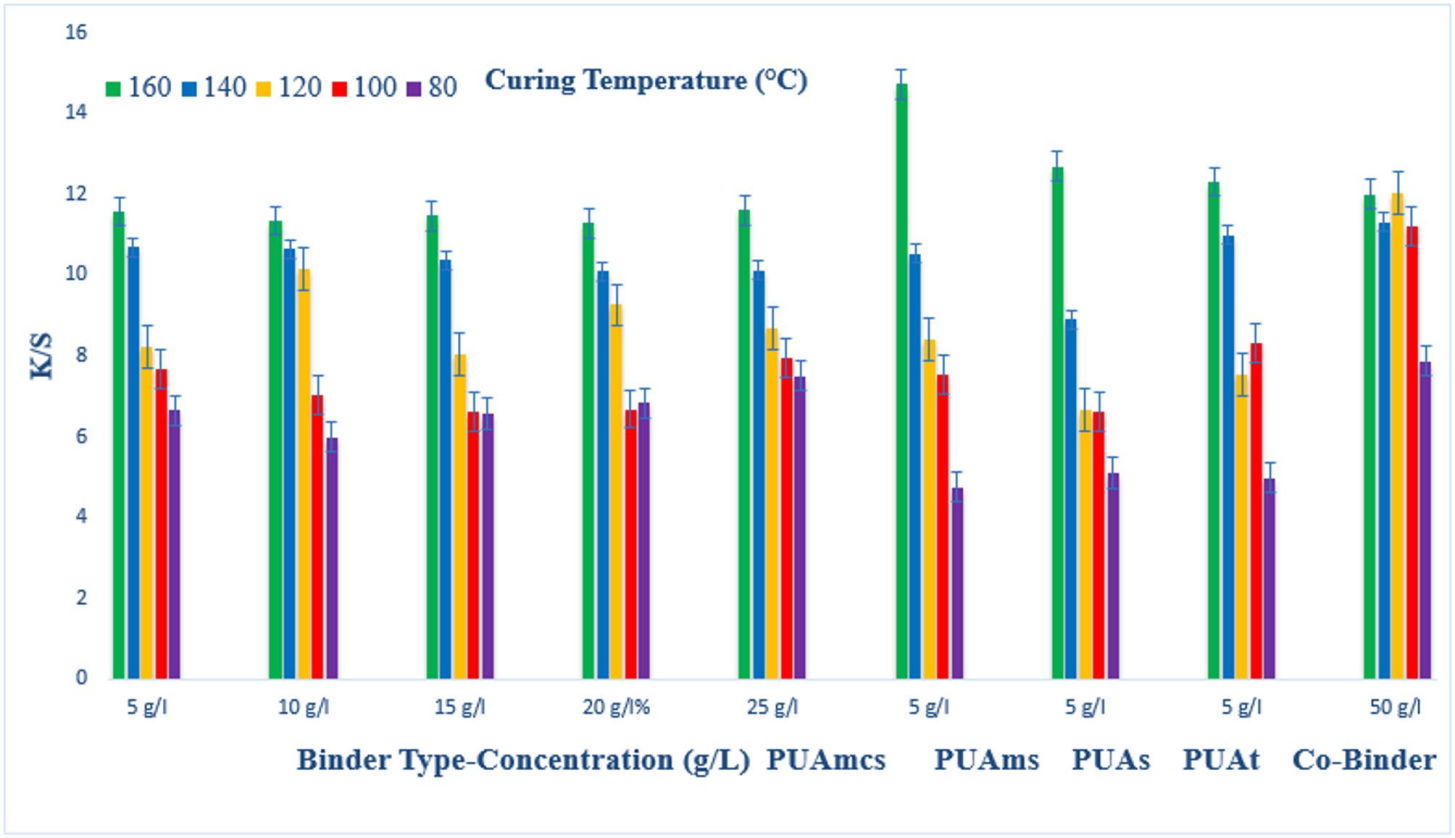
Effect of using different concentrations of binders I-IV on the K/S of printed, thermally* cured CO/PET fabrics for 6 min. *Curing temperatures 80, 100, 120, 140, and 160 °C.

**Fig. 9:**
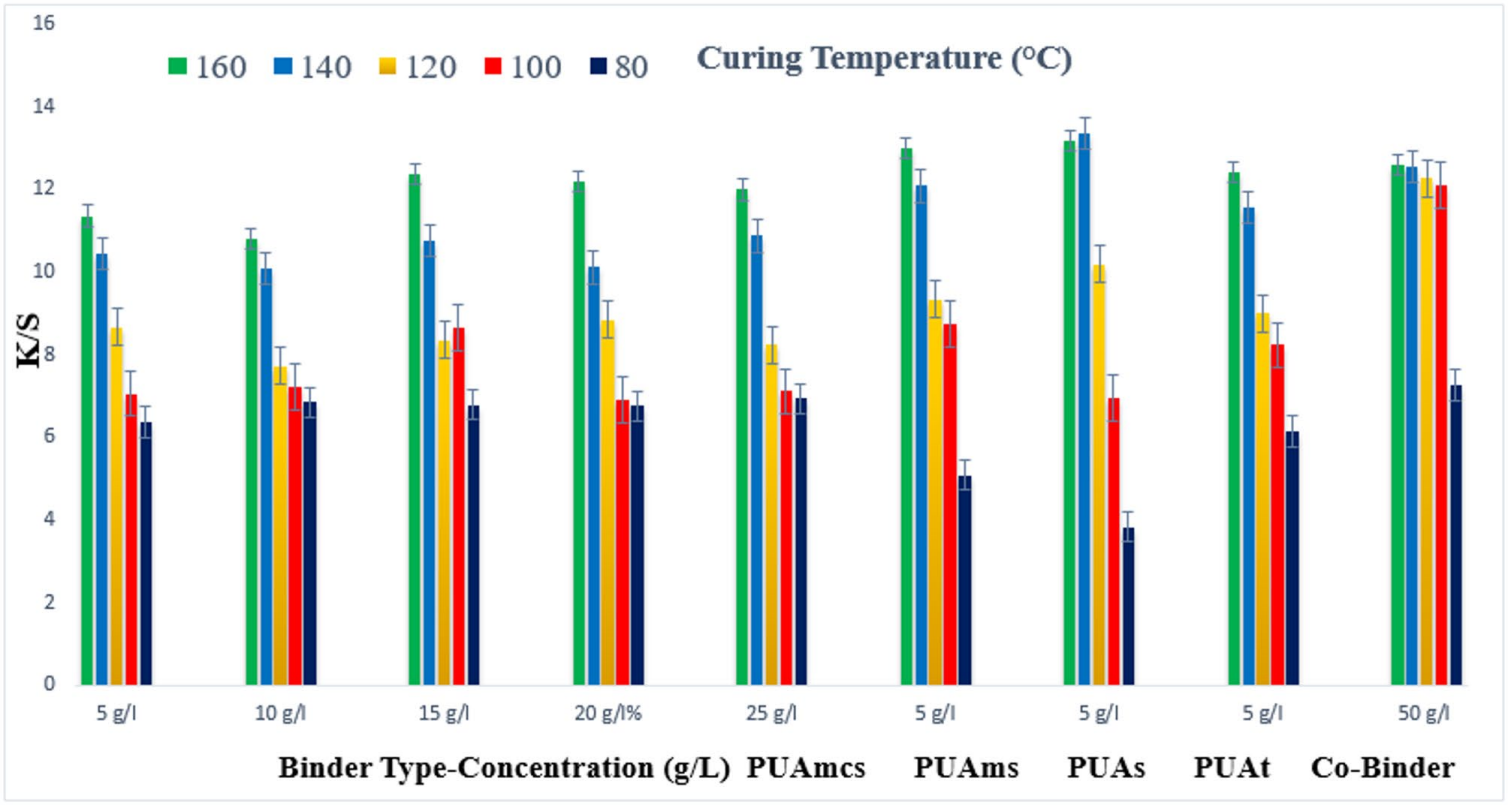
Effect of using different concentrations of binders I-IV formulation on the K/S of Printed, thermally* cured CO/PET fabrics for 8 min. *Curing temperatures 80, 100, 120, 140, and 160°.

Figures 7, 8 and 9, show the effect of using the aforementioned binder’s concentration on the K/S of the printed CO/PET fabrics. From the Figs, it is noticed that binder PUAmcs, upon using concentrations of 5 and 10 g/L in the printing paste, produce comparable K/S results. Further, an increase in the binder concentration up to 25 g/L produced no significant color enhancement, and therefore, it is not recommended. The reverse is noticed with the commercial binder, which needs higher concentrations of 50 g/L to produce CO/PET prints with comparable K/S values to those obtained using synthesized binders. This affects the economic aspects of the process.

From Figs. 7, 8 and 9, it is clear that the synthesized binders’ I-IV produced CO/PET prints with comparable K/S values, in most cases, as compared to those produced using commercial binders. Furthermore, it was observed that in some cases (using synthesized binders), higher K/S values are produced.

The effect of increasing the thermal fixation time from 4 to 8 min on the K/S values of the prints is also shown in Figs. 7, 8 and 9 respectively.

It is clear that the fixation time of 4 min. This could be considered adequate time under such conditions, and no need to prolong the time of fixation any further.

Also, it is noticed that the effect of increasing the thermal fixation temperature from 80 to 140 °C leads to an increase in the K/S, and after that, increasing the temperature to 160 °C, produced a slight increase in K/S values. This is true irrespective of either the concentration or the type of binder used. Therefore, it is also not recommended to use higher fixation temperatures over 140 °C in this case to save energy. The optimum conditions of thermal fixation could be considered as 140 °C for 4 min.

Generally speaking, binder IV PUAt seems to have produced prints with the highest K/S values as compared with the results obtained by the commercial binder and (PUAmcs, PUAms, and PUAm), in most cases. This could be attributed to the increase in the amount of vinyl groups which leads to increased binder polymerization resulting the better pigment fixation.

As shown in Fig. 7, For example, using binder concentrations of 5 g/L in the printing paste, using PUAmcs, PUAms, PUAm and PUAt, at fixation temperature 160 °C and time of fixation 4 min., the K/S values is 11.5, 11.4, 13.8 and 12 respectively, compared to the commercial binder with concentration 50 g/L in printing paste at the same condition used the K/S is 11.6.

The following illustrates the work mechanism of polyurethane acrylate as a bonding agent for developing a durable, flexible film that binds pigments to fabrics: During the curing, initiators generate free radicals. These radicals react with the carbon-carbon double bonds in the acrylate groups (C = C), initiating a chain reaction. When the acrylate groups break their double bonds, they form covalent connections with adjacent PUA molecules or other reactive species. Crosslink Formation: Heat activates thermal initiators that enhance crosslinking by allowing opened acrylate groups to bond with multiple PUA chains, resulting in the creation of a three-dimensional polymer network. This network formation, also known as crosslinking, results in a strong, cohesive film that encapsulates pigments and adheres to the textile surface. Encourage crosslinking by combining multiple PUA chains that contain open acrylate groups. This approach creates a three-dimensional polymer network capable of effectively encapsulating pigments and adhering to the textile surface. Physical adhesion and dispersion stability can enhance the durability, flexibility, and resistance to washing and abrasion of the resulting film. Additionally, the soft segments of PUA are flexible, allowing the printed film to expand with the fabric without cracking, while the hard segments provide strength^20,21^.

### Scanning electron microscopy

**Fig. 10:**
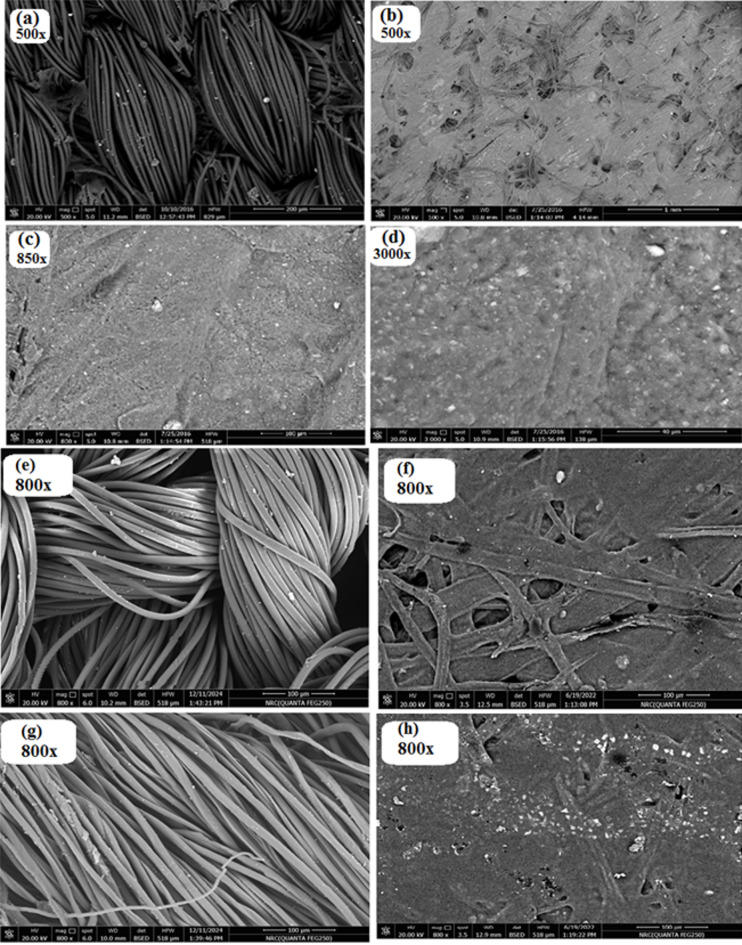
SEM images of printed cotton*, polyester**, and blends*** fabrics without and with PUAmcs binder. *(**a**) and (**b**,** c**,** d**) **(**e**) and (**f**) *** (**g**) and (**h**).

Figure 10. The a, b, c & d images represent the scanning electron microscopy of the selected printed cotton fabrics, samples printed, dried, and then cured at the recommended fixation conditions (160 °C and 4 min). After curing, the samples were washed with water to remove any unfixed pigment particles from the fabric surface. Image (a) is denoted for SEM images of pigment-printed cotton fabric by a printing paste that does not include binder (PUAmcs as an example) in its formula, while Image (b) is for the surface of the same fabric printed by a paste that provides for PUAmcs binder.

From image (a), which is, represents the surface of fabric printed with a paste containing no binder, the fabric cannot fix or hold the pigment particles and keep them inside it. While the other image (b) forms a homogeneous & continuous film that retains the pigment particles and fixes them inside it on the surface of the printed fabric. For more illustration, image(b), has been magnified to 850, and 3000 X is represented in (c) & (d) images, respectively. From these images, we can note the formation of an ordered, homogenous, and uniform film on the fabric surface.

Moreover, Images (e), (f), (g), and (h) depict blank and printed polyester fabric, as well as blank and printed blends fabric, following fixing and washing. The images demonstrate a consistent layer of printing paste applied to the fabric surface, which is also uniformly and homogeneously spread, indicating the effectiveness of the bonding agent in attaching the pigment to all of the different fabrics employed.

### Fastness properties

Tables 1, 2 and 3 show the K/S and overall fastness properties of printed cotton, polyester, and CO/PET fabrics with pigment printing pastes containing 5 g/L synthetized binders (I-IV), as well as 5 g/L Green 3GL pigment, as compared to those upon using 50 g/L commercial binder (Bercolin metal CM), respectively.

**Table 1:** The K/S and overall fastness properties of printed, cotton fabrics with pigment printing pastes containing 5 g/L synthetized binders (I-IV), as well as 5 g/L Green 3GL, as compared to those upon using 50 g/L commercial binders.

Binder type	K/S	Rubbing		Washing fastness			Perspiration					
							Acidic			Alkaline		
		Dry	Wet	St. cotton	St. wool	Alt.	St. cotton	St. wool	Alt.	St. cotton	St. wool	Alt.
PUAmcs	12.31	4	3	4	4	4-5	4-5	4-5	4	4-5	4	4-5
PUAms	11.06	4	3	4	3-4	4-5	4	4	4	4	4	4
PUAm	11.42	3-4	3	4-5	4	4-5	4	3-4	4-5	4	4	4-5
PUAt	10.98	3-4	3	4	4	4	4	4	4-5	4-5	4	4-5
Commercial binder	12.26	3	3	4-5	4	4	4	4	4	4-5	4	4-5

Curing temperature 140 °C for 4 min. St. — Staining; Alt. — Alteration.

**Table 2:** The K/S and overall fastness properties of printed, polyester fabrics with pigment printing pastes containing 5 g/L synthetized binders (I-IV), as well as 5 g/L Green 3GL, as compared to those upon using.

Binder type	K/S	Rubbing		Washing fastness			Perspiration					
							Acidic			Alkaline		
		Dry	Wet	St. cotton	St. wool	Alt.	St. cotton	St. wool	Alt.	St. cotton	St. wool	Alt.
PUAmcs	13.50	4-3	2-3	4	3-4	4	4	3-4	4-5	4	3-4	4-5
PUAms	10.03	4	2-3	4	4	4-5	4	4	4	4	4	4
PUAm	11.42	3	2-3	4	3-4	4-5	4	3-4	4	4	4	4
PUAt	10.42	4	3-4	3-4	3-4	4	4-5	4	4	4	3-4	4
Commercial binder	10.05	3	3	4-5	4	4	4	4	4	4	4	4

Curing temperature 140 °C for 4 min. St. — Staining; Alt. — Alteration.

**Table 3:** The K/S and overall fastness properties of printed, cotton/polyester blend fabrics with pigment printing pastes containing 5 g/L synthetized binders (I-IV), as well as 5 g/L Green 3GL, as compared to those upon using 50 g/L commercial binders.

Binder type	K/S	Rubbing		Washing fastness			Perspiration					
							Acidic			Alkaline		
		Dry	Wet	St. cotton	St. wool	Alt.	St. cotton	St. wool	Alt.	St. cotton	St. wool	Alt.
PUAmcs	10.88	4	2-3	4-5	4	4-5	4-5	4	4-5	4-5	4	4-5
PUAms	10.04	4	2-3	3-4	4	4	4	4	4	4	4	4
PUAm	12.97	3-4	2-3	4	3-4	4	4	4	4-5	4	4	4-5
PUAt	11.32	4	3	4	3-4	4	4	4	4	4	4	4
Commercial binder	11.1	3	2-3	4-5	4	4	4-5	4	4	4-5	4	4

Curing temperature 140 °C for 4 min. St. — Staining; Alt. — Alteration.

From the results, both the K/S and the fastness properties of the prints depend on the type of binder used, as well as the type of selected fabric. The wet rubbing fastness results were low for all polyester and CO/PET prints. However, some improvement is noticed with binder IV- PUAt as compared with the other binders used, including the commercial binder, and gives a dry rubbing range from good to very good. Still, the other binders give a dry rubbing ranging from moderate to good. But in the case of printed cotton fabrics, the wet rubbing is good for all binders used. The dry rubbing for all binders used ranged from good to very good. The washing and perspiration fastness ranged from very good to excellent for all.

The Summary, prepared binder gave printed samples with equal or higher color strength results as compared to those obtained upon using the commercial binder under the same conditions. The rubbing fastness for printed samples using the prepared binder is an improvement compared to samples printed using a commercial binder. The rubbing fastness ranged from good to very good in case of using the prepared binder and ranged from moderate to good in case of using the commercial binder, and this may be due to the prepared binder being in nano size, which leads to more fixation of the pigment onto the surface of the fabrics. Moreover, washing and perspiration fastness properties were in the range of very good to excellent for fabric printed using the prepared binders in the printed pastes.

## Conclusion

The Nano-Scale synthesized binders I-IV (PUAmcs, PUAms, PUAm, and PUAt) demonstrate notable benefits compared to commercial binders. In terms of economics, they provide equivalent or deeper color strength (K/S values) at much lower concentrations (10 g/L) compared to the conventional binder (50 g/L), minimizing the costs and increasing efficiency. On an environmental level, reduced binder concentrations and optimized curing conditions (140 °C for 4 min) reduce energy consumption (environmental footprint) by minimizing excessive resource use. Furthermore, these binders improved fastness characteristics, resulting in good to exceptional washing and perspiration fastness over all textiles and improved wet rubbing fastness, particularly for cotton prints. The above-mentioned properties can make these binders a viable and sustainable option for industrial textile printing applications. Both the washing and perspiration fastness ranged from very good to excellent for all. The dry rubbing for all binders used ranged from good to very good. The effect of increasing the thermal fixation temperature from 80 to 140 °C leads to an increase in the K/S, and after that, increasing the temperature to 160 °C, produced a slight increase in K/S values. This is true irrespective of either the concentration or the type of binder used. Binder PUAmcs seem to have produced prints with the highest K/S values as compared with the results obtained by the commercial binder and binders (PUAms, PUAm, and PUAt) in most cases. SEM micrographs clarify the formation of a homogeneous binder film on the surface of printed fabrics. The contribution of this article to future studies in the field of improvement of pigment coloration.

## Data Availability

Included in the paper.All data supporting the findings of this study are available within the paper.The datasets used and/or analyzed during the current study are available from the author in charge upon request.
